# Watching Porn, (Un)Doing Gender? Young People’s Experiences and Understandings of Online Porn

**DOI:** 10.1007/s10508-024-03076-0

**Published:** 2025-02-13

**Authors:** Claire Meehan

**Affiliations:** School of Social Sciences, University of Auckland, 10 Symonds Street, Auckland, 1010 New Zealand

**Keywords:** Porn, Gender, Young people, Sexual cultures

## Abstract

The social construction of gender and pleasure is a dominant discourse that is historically entrenched within New Zealand society. Like elsewhere, New Zealanders have seen an exponential rise in the availability and accessibility of online porn in the past few decades, yet a gap in the literature remains around how young people perform gender while engaging with porn. Drawing on qualitative small friendship group interviews with 106 cis gender young people in three New Zealand schools, I explore the ways in which young people “do” gender while watching and talking about porn. I found that the young people both conformed and diverged from traditional constructions of gendered pleasure and sexual expressions. It was clear through an analysis of these data that the structural context and dominant discourses impacted the meanings young people attached to their experiences with porn and interactions with peers about porn.

## Introduction

The past few decades have seen an exponential rise in the availability and accessibility of online porn, for adults and young people alike (Byron et al., 2021). This article explores the ways in which young people “do” gender, particularly heterosexual cisgender, while engaging with online porn. I start by exploring the relationship between gender and porn, before examining the construction of gendered sexual pleasure, a discussion on “doing gender” followed by an investigation of porn, gender and sex education. I then present findings from qualitative small friendship group interviews with school-aged young people in New Zealand, focusing first gendered norms, then acceptable pleasure, risky pleasure, before looking at resistance and disruption. Finally, I consider whether young people are (un)doing gender while watching porn. I found that the young participants both conformed and diverged from traditional constructions of gendered pleasure and sexual expressions. It was clear through an analysis of these data that the structural context and dominant discourses impacted the meanings young people attached to their experiences with porn as well as their interactions with peers about porn. I argue that sex education programmes need to address the impact of gender on young people’s experiences with porn.

It should be noted that this study draws on the terms “girls” and “boys” or “young men” and “young women” throughout this article because that is what the young people who participated in this research self-identified as. While there are many different gender identities, in this work, gender is viewed from a constructionist perspective and from a performative perspective.

### Gender and Porn

In New Zealand, the Office of Film and Literature Classification conducted an online survey with 2071 young people aged 14–17 years. Of those who had watched porn, 93% thought it was common for boys and 50% thought it was common for girls (Henry & Talbot, 2019). Frequent reasons for watching porn include curiosity, education, pleasure, and masturbation (Bale, 2011; Mulholland, 2013; Sabina et al., 2008).

Traditionally, porn has been framed as a risk to young people’s wellbeing and development (Albury, 2014; McKee, 2010). This risk discourse is highly gendered and as a result, young people, especially girls, are often excluded from discussions on porn (Scarcelli, 2015). While acknowledgment of adult women’s porn use is growing (McKeown et al., 2015), porn remains a gendered phenomenon for young people in terms of perceptions and use, in New Zealand (Henry & Talbot, 2019; Meehan, 2023) and elsewhere (Yu et al., 2021). For example, we know that young men typically watch porn more frequently then young women (Tsaliki et al., 2014) and they are more likely to be comfortable taking about their engagement with porn (Meehan, 2023). In turn, older teen boys tend to perceive porn more positively than girls and younger boys (Martellozzo et al., 2016). Heterosexual porn use has been largely normalized for young men (Mulholland, 2015) and regularly viewed positively, whereas it is often still considered negatively for girls (Meehan, 2021; Tsaliki et al., 2014). Some young women have talked about porn use as disgusting, dirty (Scarcelli, 2015) or shameful (Meehan, 2022), while others have reported using porn to relieve stress (Attwood et al., 2018), identify their desires (Bhana, 2008), as a source of information (Ramlagun, 2012) and for fun (Attwood et al., 2018).

While there is an abundance of literature on young people’s gender—often in a binary sense of girls and boys, there is gap around performance and performativity of gender. Considered a set of cultural practices (Attwood, 2005), porn is an interesting space to explore young people’s gender performativity. This article seeks to explore the ways in which young people perform gender while engaging with porn, both watching and discussing porn, and questions the performative nature of these phenomena.

### The Construction of Gendered Sexual Pleasure

Scholars continue to offer different understandings of sex and gender. Many construct sex and gender in relation to the ways society labels people male or female. Sex is often coupled with biology, for example, what a person is assigned at birth. Gender is more closely related to cultural influences (Muehlenhard & Peterson, 2011). The social construction of gendered sexual pleasure is a dominant discourse that is historically entrenched within New Zealand society and elsewhere. Prior to the 1960s, gender was considered as what constituted masculine and feminine, the conceptualisation of two social groups—“men” and “women” (Robinson & Richardson, 2015), where men held power over women. Traditional gendered understandings prioritize men’s sexual pleasure and frequently position women as the means of achieving that pleasure (Brown et al., 2018). In this sense, sex is routinely defined as a heterosexual act, encompassing vaginal intercourse, and resulting in male orgasm and ejaculation (Meehan, 2021). Orgasm is frequently considered to be the pinnacle of sexual experience (Allen, 2003), of “sex done well.”

Research has demonstrated that the greatest disparity in orgasm is between cis (those who identify with the gender they were assigned at birth) men and cis women (Harvey et al., 2023). This has been explained for two main reasons, first, sex often ends with male ejaculation (Allen & Carmody, 2012). Second, both men and women report male orgasm is an essential part of sex, without which sex is considering to be “incomplete” (Andrejek et al., 2022). While male orgasms are considered a normative occurrence, women’s orgasms are thought to be of lesser importance and less central to the overall experience. During heterosexual sex, penetrative sex alone is unlikely to result in a female orgasm, and the male orgasm is frequently privileged above the woman’s desire (Braun et al., 2003). In a similar vein, depiction of male orgasm in porn is much more frequent than female orgasm and often includes visible ejaculation. This ejaculation, colloquially known as “the cum-shot” or “the money shot,” usually indicates the cumulation of the scene. This has been understood in dual ways, to confirm male pleasure and its authenticity in the film or to symbolically debase women (Shor, 2023).

In this way, sexual activity is thought of in terms of an active *male* subject and passive *female* object (Jackson, 1996, p. 23, emphases added). Traditional understandings of pleasure prioritize men and frequently position women as the means of achieving that pleasure (Brown et al., 2018). Gendered cultural scripts about sexual desire assume that men have strong sexual desire, whereas women have a naturally weak desire for sex (Rubin et al., 2019). Scholarship continuously highlights the complexity of both young men’s and young women’s understanding of sexual activity (e.g., Meehan, 2023), yet the conceptualisation of men as sexually aggressive and women as sexually submissive has been influential (Gavey, 2018). Through the male gaze, young women are expected to remain “pure,” while their male counterparts are encouraged to be sexually liberated (Vickery & Everbach, 2010). Young women who are seen to be sexually knowing risk being blamed or slut shamed (Ringrose & Renold, 2014). This discourse has created cultural contexts which have meant that discussions around young people’s, especially young women’s, sexual desire have remained absent or vilified (Ringrose, 2012).

### Doing Gender

This study builds on Butler’s (1990) adaption of West and Zimmerman’s (1987) concept of “doing gender” to gain insights into how young people understand porn. Within this construction, people routinely perform gender by doing and enact gender performativity by bringing regulatory discourses into being, meaning to embody or “live” gendered norms which are dominant in society. It is important first to understand the difference between performance and performativity. Put simply, performance accepts a pre-existing subject, whereas performativity contests the very existence of the subject. Butler (1990) first suggested that gender was performative—it is conceptualized and understood through the repetition of certain acts, rather than something that is inscribed on the body. These acts are a practice of improvisation within a scene of societal, familial, and legal constraints to the extent that they appear a “natural kind of being” (Butler, 1990, p. 33). Therefore, gender is a learned behavior within a highly rigid regulatory framework of heteronormativity, in the form of language, discourse, and repetition. For Butler, there is no gender identity outside of performance, people do not have gender, they accomplish it through performance, which is an endless process—it must be consistently reaffirmed and publicly displayed by performing particular acts in accordance with the cultural norms of masculinity and femininity (Cameron, 1997). In this way, gender is dynamic and fluid, it is performed according to a multitude contexts and social roles, culturally policed and enforced by punitive rules. Femininity and masculinity are not what we are, but the effects we produce due to specific things we do. They are not stationary but plural and conflicting notions (Weatherall, 2002, p. 6). Gender identity is an active and perpetual process, that is inescapable.

Central to the concept of doing gender is the link between gendered norms and power relations—the ways in which masculinities and femininities should be performed. There are risks associated with non-normative performance of gender which means that individuals behave in ways which assess the risks associated with this performance to understand how their behaviors will be classified (Harvey et al., 2023). While this article uses gender performance and performativity as a framework, there are limitations to this approach. Both sex and gender are often considered in binaries which exclude the lived experiences of non-cis gendered people (van Anders, 2015).

While the focus of this article is on gender, multiple frameworks have been put forward to aid our understanding of the relationship between gender and sexuality. Some scholars have posed a direct causal connection between the two—that is, gender constitutes sexuality and sexuality constitutes gender (MacKinnon, 1982), while others have separated the two as distinct but interrelated realms of social practice (Rubin, 2002; Rubin & Butler, 1994). Still others see them as analytically separable and closely interrelated, according priority to one over the other (Jackson, 2005). Butler (1990) suggests that we investigate the “complex interimplication” of gender and sexuality. By situating porn use within an approach that sees gender/sexuality as distinct, yet “all erotically significant aspects of social life and social being—desires, practices, relationships and identities’ in addition to “a person’s sexual orientation, interests, acts, expressions, and/or experiences” (Sedgwick, 1990, p. 27), we can begin to explore how porn use may function in different ways, both public and private, in relation to young people’s understandings of gender, sexuality, sex, sexual orientations and identities (Smith et al., 2018).

### Porn, Gender, and Sex Education

Scholars such as Smith (2013) found that porn may be used as a resource for young people to develop their values and beliefs about sex, gender and sexuality and sexual orientation and identity, especially when they perceive their sex education to be inadequate. In more recent research, Dawson et al. (2019) discovered that perceptions of sex education were irrelevant, instead young people are potentially motivated be motivated to watch porn for different reasons, and it may only contribute to the numerous ways they learn about sex, gender and sexuality.

Balanced discussions about porn remain largely absent in sex education in New Zealand schools, due to the nature of assumptions about young people’s sexuality as well as other practical reasons (Meehan, 2023) and sexuality education is delivered through a risk-based approach. In doing so, Albury (2014) contends that sex education likely fails to adequately engage with the gendered and cis-heteronormative dimensions of young people’s sexual cultures. Albury recommends that meaningful sex education would explore why young people find porn arousing, desirable and pleasurable to can be a means to formulate and express their subjectivities.

## Method

### Participants

Small friendship group interviews were conducted in 2018–2019 with 106 self-selecting young people, aged 12–16, in three participating schools in New Zealand. Participants were asked to selected their gender identity, but not their sexuality/ sexual identity. Small friendship group interviews of established friends in the same year group allowed many of the benefits of a focus group (Krueger, 2014), but overcame some of the limitations of a standard sized focus group, such as the participants not knowing or liking each other (Allen, 2006). It was hoped that a comfortable small group setting with established friends would be more conducive to providing deeper insights and respecting confidentiality (Table 1).

**Table 1:** Characteristics of sample

School	Number of participants	Number of groups	Gender
1	35	11	22 girls, 13 boys
2	31	8	Boys
3	40	8	Girls

### Procedure

Young people were recruited from one rural co-ed—35 participants), and two urban single sex (one male—40 participants, one female—31 participants) schools and participated in 27 small friendship interview groups. Before each session I provided the groups with an overview of the study, what the session would entail, confidentiality and the importance of keeping what is said within the group. I talked about the ethical implications and reiterated that participants did not have to answer any questions and they could leave at any time without giving a reason. I provided refreshments and the groups began In each of the groups we started with an icebreaker—sharing participants’ names or what they wished to be called, their favorite TV show, and how they knew each other, followed by a scenario on porn to open discussion then focused on loose topic areas, rather than pre-set questions. This narrative format allowed the participants to set the agenda and focus on areas that were of most interest to them. Groups consisted of between two and six participants and lasted on average 80 min. The participants and I were the only people present at the group interviews which were held in a classroom at each school. Young people were given the option of having a one-on-one interview instead of participating in a small friendship group but no one opted to do so.

### Analysis

Data were recorded and transcribed by a professional transcriber and I verified the transcripts. Drawing on Braun and Clarke’s (2006) six step thematic analysis framework. The author coded and analyzed the data using an iterative and inductive approach, which enabled the creation of a codebook, defined by new insights and relationships in the data. While statistical methods of inter-rater reliability were not appropriate given there was one coder, analytic memos, created after each small friendship group interview aided with contextualizing the analysis of transcriptions, which provided a means of reflection and review of codes I considered how shared meanings, reflexive interpretations and interpersonal experiences shaped young people’s experiences, understandings and meanings about porn to generate codes, sub-codes, themes and organizing themes until I reached data saturation—where no new concepts were being identified in the data. These themes were then identified, reviewed, further defined and situated within the scholarly literature. I note throughout the following section the frequency of which themes arose by stating whether it was raised by most, some or a few participants.

## Results

It should be noted that porn is an umbrella term which encompasses the above but also includes a diverse range of genres, such as alt, kink, feminist, queer, ethical and exploitative porn. McKee et al. (2020) convened a Delphi panel of 38 leading porn researchers from a wide range of disciplines. They found that there was no agreement on a single definition of porn. Basically, porn is notoriously difficult to define. It is not one thing. In this study the young participants were mostly consuming mainstream free online content from sites such as YouPorn and PornHub and the discussions center around this particular genre of porn.

To explore the ways in which young people “do” gender while watching and talking about porn, the findings are organized as follows: (1) gendered norms, (2) acceptable pleasure, (3) risky pleasure, and (4) resistance and disruption. These categories resulted from gendered themes which were common within the research. Excerpts below are taken from young people, who are referred to by pseudonyms such as “Sarah” throughout. Group sizes ranged from three to six participants and groups are categorized, recognizing that they are not mutually exclusive, as younger teens (12–13 years), mid-teens (13–15 years) and older teens (15–16 years). The majority of groups represented below were single sex (groups A, E, R, T were girls, groups D, G, L, P were boys), and groups C and S were mixed sex.

### Gendered Norms

Young people’s response to who watched porn and why were overwhelmingly gendered. Both boys and girls felt that it was normal for most young men to watch porn. Reasons for boys watching included “masturbation,” “boredom,” “curiosity,” “fun” and “to relax.” Others watched it as a source of informal education “to see things and learn how to do them” [Dan] and “to see what I like, you know, sexually” [Liam]. In alignment with a gendered perspective on porn use, these statements also speak to its use as an educational resource as well as opportunity to see and learn about orientation-consistent sexuality, and for young men to clarify their self-identification of their sexual orientation and/ or sexual preferences.

Most boys felt there use of porn was normal and appropriate for their age and stage of life and they considered their use to be harmless. Many of the boys considered their experiences with porn to be positive, porn was “fantasy” and different from “real life.” In this way, they positioned themselves as critical consumers of porn. Those who watched porn often did so regardless of whether they were in a relationship but would watch less if they were sexually active or in a sexual relationship, indicating that sex within a relationship held higher value than masturbation via porn.

The majority of participants felt that most girls didn’t watch porn and those who did didn’t watch as much as boys. Most of the boys reported thinking that the majority of girls didn’t watch porn as they wouldn’t be aroused by it, didn’t masturbate, or “could have sex whenever they wanted” [Dan], suggesting that girls were considered to be sexual gatekeepers. Both young men and women agreed that porn use for girls was taboo and carried a certain amount of shame and stigma. Porn use for young women was generally considered to be “too much” and for “certain girls” [Sarah], “It’s different for girls. You’d have to be desperate to watch it” [Nora]. The implication here is that girls who watched porn are too sexual, promiscuous or desperate. These “certain girls” were identified and othered as not conforming to the bonds of acceptable, or normative, femininity (Ramlagun, 2012).

In the following conversation, the girls discuss the type of porn that they believe women watch before Emma reports that she has watched porn.

Claire: Do women watch porn.

Emma: Yeah, definitely not as much, but I think it’s coming more kind of mainstream for women to actually watch porn.

Erin: But women are more interested in actually like literature and stuff. Sorry, I’ve read this somewhere.

Emily: That’s what I've heard at least, because… I feel like, okay, so porn is typically like quite fake, and stereotypical, and it’s like built around the male fantasy. Whereas, fiction was kind of like women’s escape for a while

Emma: I have watched it. I feel like porn is kind of developing as an industry, and I feel like they’re trying to open it up. This is the first time I’ve talked about it – are you [looks at peers] surprised?

Here, we can see that the juxtaposition of expressions of sexual interest, for men it is usually positive, whereas it negatively impacts young women. In the conversation above Emily reveals that (free, online) porn is fake and “built around male fantasy,” thus not reflective of female pleasure. Noting the gendered orgasm gap in mainstream heterosexual porn, it is unsurprising that girls didn’t feel that they were represented. Boys were performing gender through watching porn and feeling able to discuss it openly with their friends and even in the friendship group interviews for this study. This was considered a normalized, accepted and almost expected part of being a teenage boy. Girls were also performing gender in that the majority did not disclose porn use and it was considered unnecessary by both young men and women. Girls were not considered to be actively sexual in the same way, they were not seen to act upon any hormonal or sexual urges. If they did watch porn, it wasn’t common practice for them to discuss with their peers. As Emma points out, this was the first time she’d ever talked about watching porn.

### Acceptable Pleasure

Pleasurable engagement with porn was both overt and covert, and it was reinforced by traditional gendered narratives. Both watching and talking about porn was constructed by both young men and young women as part of boys’ heterosexual masculinity. For boys, pleasure in terms of both watching porn and discussing it was overt. Direct pleasure was gained through sexual gratification and masturbation while directly engaging with porn.

Ciara: Porn’s more like kind of a boy thing because guys are hornier. I think most guys do it

Cam:It’s kind of normal, because it's basically like a pleasure.

Ciara:And they talk about it all the time.

Colin: Speaking from hostel point of view, we all talk about sex and porn things at hostel. We usually talk about it amongst the boys. But I don’t know about girls.

Ciara: I don’t remember ever really having a discussion with any of my friends about porn.

Cam: Yeah, I feel like girls wouldn’t tell anyone because they’d judge them coz it’s quite, it’s not weird but it is weird for girls

As is evident from the conversation in the mixed group above, and aligning with many other participants’ reports, porn was considered as something created for men’s pleasure, and it was viewed as a rite of passage for many young men to enjoy watching porn. Here we can see instances in the young people’s discussions where they made slippages between sex—biology—and gender—cultural influences. Young men were thought to be much more interested in sex than women, and both boys and girls cited biological explanations as to why boys need to watch porn more frequently for a release to satisfy their sexual urges “they can’t help when they get a boner” [Sarah]. In this way porn use was framed as something naturally connected to the essence of being a man (Weeks, 1985). While boys were described as “hornier” than girls, thus cementing their sexual urges as biological and persuasive, girls were simultaneously limited in their ability to use porn as part of creating and producing their sexual identities. By considering porn use by girls as “weird” or “desperate,” the implication is that most girls are not sexual beings. Again, girls were reduced to being situated within an acceptable femininity, in terms of both watching or discussing porn—two areas which boys appeared to derive pleasure.

Sean: If someone was to say, “I’ve watched it.” It’s immaterial, it’s a oncer or more or whatever. It sometimes get to the stage where people pat you on the back and say, “Oh yeah you do that and stuff?” It’s a very normalized thing. For a lot of boys it’s kind of daily routine and stuff; it’s like a pat on the back become a mission accomplished thing.

Alongside the immediate pleasure of sexual gratification, there was a secondary, more indirect form of pleasure. This was obtained through the opportunities porn presented for homosocial bonding with peers, by exchanging anecdotes and shared experiences, as Sean notes “like a pat on the back.” Discussing porn provided boys an opportunity for homosocial bonding. In this sense, homosociality facilitates a social dynamic which upholds hegemonic masculinity.

In the conversation below, girls reported some of their perceptions of common themes in porn, such as lesbian, milf, gang bang, as content created for men’s desires and not necessarily a palatable space for women. These girls drew on discourses that were underpinned by dominant meanings about gendered sexual expression. These dominant meanings, which have been inscribed over time, frame porn as a male arena created by and for men.

Rachel: I don’t know, it's just like it's doesn’t seem like something you’d picture; like us, as women, it doesn’t seem like something we’d do ourselves so we wouldn’t imagine it as a girl watching it.

Rebecca: [laughs] like milfs or anal or lesbians.

Rachel: Or when there’s all these men and only one woman.

They felt this content was uninteresting or unpleasant for most women. When young women did use porn, there was usually a justification that didn’t include pleasure, for example seeking information of to find out “why the guys like it so much” [Laura]. For those who did derive pleasure through watching porn, this pleasure was problematized.

### Risky Pleasure

Pleasure from porn was much more covert for young female participants. The majority of young women denied any pleasurable experiences with porn and some questioned that it was possible for women to get pleasure from watching porn. There were concerns with pornographic acts as being violent, “not nice for the women” [Gemma] and general concerns about the treatment of female performers, “why is it an obsession for guys to tie girls up and make them choke. Why do they like that?” [Jane]. Unlike the boys’ views that porn was “fantasy” and different from “real life,” girls found fantasy and real life hard to separate.

In this research only a small group of older-teen girls reported watching porn for pleasure, and even then it was framed as a “dirty pleasure.” In the conversation with Gina and Gemma, both 16 years old, below, it is clear that watching porn is both pleasurable but inherently risky—they risk feeling like they are objectifying other women by watching porn, as well as potential reputational risk should anyone find out.

Gemma: Yeah, but a dirty pleasure

Claire: Why dirty?

Gemma: I think in some ways it is because when you hear about the harm towards women sometimes. You feel like you shouldn’t do it

Gina:Yeah, it would be kind of weird “cause it’s like fulfilling your needs but you know it’s objectifying women

Gemma:I don’t want people to think I’m slutty either

Gina:If girls go around and watch a lot of porn; they’d be seen as a slut and a really negative thing. Whereas, if you’re a guy you’ll be seen as a legend, and it’s quite the opposite

Gemma:Girls are scared that they will get judged but every guy is just super open about it. For the girls it’s traditional to keep that to yourself I guess, but for guys it’s commonly talked about. It’s an open subject, but I think for girls they find it a bit more traditional to keep it secretive.

Gina:Yeah, it's like double standards and it doesn’t make sense

In the excerpts above we can see some of the complexities around young women’s porn viewing. For these participants we can see how they grapple with the tensions around the harm toward female porn performers and objection of women juxtaposed with the fulfillment of sexual needs. Gemma’s comment indicates that girls tend to be “more traditional,” potentially meaning more passive or less sexually desiring than their male counterparts. As a result, girls are more likely to be “secretive” about porn as they risk judgment or reputational damage for being seen as a slut. Being secretive is a way to avoid shame, but ultimately is a means of policing women’s sexuality.

### Resistance and Disruption

Despite the prevalence of these constructions of young people’s sexuality, there were alternative narratives of how both boys and girls positioned themselves as sexual subjects. As a means of resistance and subversion to dominant heteronormative discourses about sexuality, these young people gave other meanings to their sexual encounters and experiences. Moving away from the idea that masculinity entails natural urges for sexual gratification, a number of boys in this study reported refraining from porn for several reasons. Some found it distasteful or uncomfortable, while others had concerns for the female performers, “it’s not always very nice for the women” [Paul]. Some of these young men expressed the desire to be in a relationship and have sex as part of that, “I’ve got a girlfriend, so I don’t really feel like I need porn” [Sam]. Several felt that porn was too focused on just the sexual activity, which made it unrealistic and undesirable. Other boys reported avoiding porn due to concerns around the potential for addiction, religious or cultural reasons, and a few had concerns about people they cared for finding out. Some of these conversations were bound conventions of traditional masculinity, especially around addiction where the perceived lack of self-control was linked to not adhering to a respectable masculinity. Others, like the one below, were more nuanced in terms of rejecting masculinity by not enjoying porn or feeling shame after consuming it and questioning dominant discourses.

Gavin: Sometimes you just keep going until you find something and then after you’re in a shame hole

Claire: How does that make you feel?

Gavin: Shit, but that’s what I was doing, and I didn’t want to, so I stopped.

Claire:Was it hard to stop?

Gavin:Yeah, because it’s something that a lot of guys do.

In one group we talked about different types of porn beyond the mainstream free content in PornHub and YouPorn. For some boys, they engaged with porn that wasn’t heterosexual—“like you can watch gay porn, and it's like you don’t have to have sex with this person but you can see what it’s like” [Chris], didn’t depict certain acts “I don’t like watching gang bangs” [Tony], or didn’t display human bodies, “I know heaps of boys that are into hentai” [Peter]. Some of these acts of resistance also spoke to the nature of dominant discourses within schools as well as sex education, both of which have been long critiqued for excluding same-sex sexuality as well as disavowing opportunities for same-sex attracted students to ask questions without becoming ostracized (Kubicek et al., 2010). In these ways, several boys drew on discourses that rejected dominant meanings about sexual desire and expressions. In two of these groups, the discussions themselves were less performative. As usual, the boys appeared open and honest but seemed to be less concerned with failing to measure up to their peers’ standards of heterosexual masculinity. Several of the boys actively embraced this form of gender deviance as pushback to the common narratives that they received at school—“they tell us to stay away from porn like we’re all watching it, but what if you just don’t anyway” [Peter].

While most girls recognized and discussed the gender double standard, some of the young woman, particularly in the older teen groups, created discourses that resisted dominant meanings about girls’ sexual expressions. Watching porn was often framed in terms of information seeking, and thus not erotic, or as a pleasurable experience but in conflict with their cultural beliefs and attitudes. In one group Anna discussed the importance of porn as a pleasurable pursuit for women.

Anna: Don’t judge me but I love porn!

Aria: I can’t believe you said that!

Anna: I don’t care if people think I’m a slut. I think girls should be able to watch porn like guys. I think that society is definitely changing to involve women, and not oppress women anymore, but it’s gonna take a long time to get there. A very long time.

The short excerpt above shows the tensions in Anna’s conversation about porn. Her “don’t judge me” statement prefaces her admission that she “loves porn.” As previously noted, women’s subject positions are often regulated by dominant discourses which conceptualize them as less desiring, and more passive than their male counterparts. Yet, for some young women, there was pleasure in sexual expressions such as watching porn.

## Discussion

This research sought to explore the ways in which young people “do” gender, particularly heterosexual cis gender, while engaging with online porn. It was clear that porn use is governed by gendered norms and expectations. For the young people in this study, porn viewing was normalized and considered to be a part of boys heterosexual masculinity. This is reflective of international trends (see Dawson et al., 2020; Donevan & Mattebo, 2017; Pirrone et al., 2022).

A juxtaposition of sexual interest between girls and boys was evident in the data. Common responses from boys as to why girls don’t watch porn focused around lack of sexual prowess or believing that girls don’t masturbate. This is unsurprising as topics such as masturbation and other forms of sexual pleasure are rarely (if at all) mentioned in sexuality education research or education itself (Ingham, 2014). Girls’ lack of sexual activity is picked up by Sarah and Nora who report that “certain girls” who are “desperate” watch porn. The implication here is that girls who watched porn are too sexually promiscuous. These were identified and othered as not conforming to the bonds of normative femininity. Likewise, Hale and Ojeda (2018) noted that attaining, and retaining, acceptable femininity requires constant attention and affirmation. They stated that “with every performance, gender is asserted, interpreted and marked according to its subject and context” (p. 319).

Some of the young women who recognized that more women were engaging with porn spoke about the types of porn they likely used, such as literature. This was explained as the usual platforms for providing free, mainstream porn were “built around male fantasy.” Zebroff (2023) examined the differences in duration and location of erotic touch in mainstream heterosexual porn. She found that women received significantly less genital stimulation than male performers and did the most non-reciprocal genital touch, ending with visible male climax. In this way porn normalizes dominant discourses that men’s sexual touch and pleasure are more important than women’s. Parvez (2006) in her study with women on their experiences with porn, found that a key aspect of enjoyable porn use is authenticity, which she defined as sincere expression of sexual pleasure. Given the focus on male pleasure and orgasm, mainstream porn may be perceived as inauthentic for young women. Erin notes that she’s read that “more women are actually interested in literature” which perhaps serves as a less visual, and thus more palatable, site of sexual exploration for women.

When asked why people watch porn, the most common response was pleasure. Yet, for the young participants, pleasure was gendered, complex, nuanced and potentially risky. For young men, porn was a source of both direct and indirect pleasure. Direct pleasure was achieved through porn viewing, masturbation and orgasm. It was justified biologically and hormonally as young men being “hornier” than young women, not in control of their sexual urges—not able to help being sexually aroused or “getting a boner,” as Sarah notes. Porn also provided a source of indirect pleasure through bonding with peers.

Homosociality describes and defines social bonds between persons of the same sex. Frequently used in masculinities studies, it has been defined as a mechanism and social dynamic that explains the maintenance of hegemonic masculinity (Hammarén & Thomas Johansson, 2014). By discussing porn with their friends, boys were demonstrating that they had the correct hegemonic masculine identity (Roberts et al., 2020), a “pat on the back” among friends. Men bond through a culture of watching women—“whereby the gaze of male sexual desire is used to actively display hetero-sexual masculinity for the benefit of a complicit male audience” (Thurnell-Read, 2012, p. 252). Young male participants were open about watching porn, even in the mixed friendship group interview. Discussing watching porn and even sharing clips on their phones became a form of masculine currency (Kalish & Kimmel, 2011) in their homosocial environments. This aligns with Toder and Barak-Brandes’ (2022) findings that cultural capital is acquired through access to particular forms of porn related cultural knowledge.

Interviews with boys revealed glimpses of this indirect pleasure. Goffman (1967) noted that the interactions in focus group interviews, or in this case small friendship group interviews, produce more than just “talk”: rather, these conversations are the means by which social identities and knowledge are constituted and sustained. Farnsworth and Boon (2010) furthered this by advising that the group’s dynamics and interactions help to illuminate what is often unspoken but apprehended by participants. Numerous boys in the various groups joked about porn, discussing their use in an open way with little hesitation. Here, the conversation was not only about masculinity, but it was also a performance of masculinity—the narrative was constituting people as gendered subjects (Cameron, 1997).

In a similar way to some of the ways young men were “doing masculinity” in the groups, young women were “doing femininity” through their discussions about the unpalatability of porn. If girls did use porn, pleasure was covert and denied by most girls, or framed as dirty. Girl’s discussions of pleasure were full of tensions and complexities. Gemma talked about the harms toward women and Jane questioned why guys like watching girls being choked—for these girls porn wasn’t harmless. Sexual choking in porn has become more frequently depicted, arguably commonplace, in recent years (DeKeseredy & Hall-Sanchez, 2017). Portrayals of choking in porn are highly gendered with men choking women (Vera-Gray et al., 2021), reflective of other forms of sexual aggression. Their observations of the female performers” (mis)treatment can be understood through Marques’ (2014) concept of vicarious viewing. That is, the viewer’s ability to imagine themselves in that situation. Marques (2018) conceptualizes that the female spectatorship or gaze involved a multi-level engagement with porn, including emotional connectedness between the performers and aesthetic similarities with the female performers. As Gina noted, this creates a tension between objectifying women and fulfilling the sexual needs of women.

Research has demonstrated how women respond to porn in various and complex ways. The concept of getting sexual gratification from porn but feeling tension in terms of their womanhood isn’t a new concept. For Ciclitira (2004), “women’s views, experiences, and feelings about pornography are variegated, individual, and complex” (p. 194). In her UK-based research with women aged 23–52 years she found that porn was used as sex education, for construction of erotic fantasy, or permission to be more sexually active. The prevailing view amongst female participants was that porn is a male-centered, anti-women activity which led her participants to experience negative emotions such as “guilt, shame and confusion about their own sexuality.” More recently, Paasonen’s (2021) Finish respondents reported enjoying scenarios depicting rough sex, domination, humiliation and non-consent, while distinguishing these preferences from their own ethical, political, and moral values. Unlike boys, girls’ fantasies weren’t free from the relations of social power (Butler, 2000), they were not considered “harmless.”

In saying this, we know that the power of these dominant discourses is not insurmountable. Foucault (1980) advised that where “there’s a relation of power there’s a possibility of resistance” (p.13). In different ways, some of the narratives offered by young people in this research were challenge these dominant gendered and sexual discourses. For example, some young men did not engage with porn for various reasons or young women who did. There were a number of explanations that young men in this study gave for not using porn, including concerns for female performers, porn being unrealistic, feelings of shame, being in a relationship or having someone they cared about finding out. While research suggests a high prevalence of porn use among boys (Henry & Talbot, 2019; Scarcelli, 2015), it is important to be mindful that young men aren’t monolithic. In Sweden, Johansson and Hammarèn (2007) found positive attitudes, ambivalence, neutrality, and negative attitudes toward porn from young men. In the USA, Hoagland et al. (2023) found that reasons why young men morally objected to porn included culture, shame and relationship status. While some of the discussions of resistance by boys were within the boundaries of traditional masculinity, others rejected dominant discourses about sexual desire. This resistance complicates dominant narratives about gender and masculine (hetero)sexuality.

By exploring the effects of resistance on its audience, we may be able to recognize the circumstances under which those actions alter normative conceptions, and how they may facilitate change (Deutsch, 2007). In this study, we can see porn as a site of resistance for some young people, whereby the participant’s individual acts can go beyond their individual identity to create possibilities for others. For example, young women who witness others deriving pleasure from porn may feel able to do so themselves, or boys who witness others challenging heteronormative discussions that certain porn is a rite of passage for young men may feel able to resist rigid masculine norms.

Several girls in this study created discourses that resisted dominant meanings about girls’ sexual expressions—information seeking, and thus not erotic, or as a pleasurable experience but in conflict with their cultural beliefs and attitudes—but as a pleasurable pursuit for women. For example, Anna stated she loves porn and knows this is not socially acceptable and calls it out with courage. The gendered and sexual double standard in which young women live produces a need to stay within the bounds of acceptable femininity.

Dominant discourses conceptualise woman as less desiring, and more passive than men. “Softer” forms of porn, such as literature as Erin notes, may be deemed more feminine, yet as Paasonen (2021) found, for some, “porn for women” which centred around relationships, intimacy and female desire was considered boring. Nevertheless, Anna recognized the tensions by stating “don’t judge me” before admitting that she “loves porn.” Here, Anna demonstrated how she has adopted the attitude that she doesn’t care about being thought of as a slut for doing so. In this way, she simultaneously resisted the slut label—by asking not to be judged—and accommodated it, by stating that she doesn’t care. As a signifier, the term slut has immense force in a wider socio-historical meanings of femininity (Ringrose & Renold, 2014). Gender performativity creates conceptual understandings of what is normative, and thus desirable, and problematizes alternative subject positions. By re-signifying the slut label, Anna is establishing resistance by disputing and attempting to transform this gendered norm. In doing so, she recognizes that these changes—such as making more subject positions available to women—are going to take time.

It is clear from the data that the young participants were both performing gender, they attributed meanings to the experiences of gender performance, and gender was also performative for them. Hegemonic masculinity is a system of power that sustains gendered inequality through the support it garners from dominant discourses which we have been conditioned to accept without question. These young men drew on dominant discourses of male heterosexuality in the development of their sexual subjectivities. Watching porn was a means of being (and being seen as) sexually active or assertive, part of being a man. These subject positions created a context where they could be sexual desiring, emotionally detached and “masculine” (Allen, 2005). Language and discourse around heterosexual masculinity allowed these young men opportunities to negotiate and inhabit their social environments in ways that were inaccessible for young women. The young men used these opportunities to assume subject positions which were in opposition to traditional constructions of female sexual identities.

The ways in which young people “do” gender while engaging with online porn undoubtedly has implications for sex education. A common assumption of the gendered consumption of porn is that men watch and enjoy porn and women don’t. While findings from this study partly support this, it is important to understand how young people both do and resist gender. Presently, most sex education programmes are focused on protecting young people from porn, and discussions of pleasure remain absent (Albury, 2014). In addition, they do little to address the impacts of established gendered norms, including hegemonic masculinity, or provide alternatives to the hegemonic language available to young people (Haste, 2013). While the age of first porn encounters varies, research suggest that many young people are watching porn before they have received school-based sex education (Dawson et al., 2020). This research suggests the need for sex education to recognize that some young people watch porn and some don’t, regardless of gender. In moving away from a homogonous gender-based approach, sex education can begin the tackle the implications of the gendered implications of porn use or non-use. Likewise, an approach which interrogates the performative nature of gender, alongside discourses of maleness and femaleness as normative may provide a more inclusive space for young people who do not identify with a particular, or any, gender.

### Limitations and Avenues for Further Research

This research explored the ways cis gendered young people did gender while engaging with porn. It is important to recognize that the majority of participants in this study self-identified as cis gendered and heterosexual. As a result, non-cis-heteronormative subject positions may been underrepresented. Research suggests that heterosexual cis gendered young people may have differences in first experiences and prevalence of porn use than those who are trans or nonbinary (Bőthe et al., 2020). Doing gender—engaging in gender displays that align with gender roles and expectations—has been critiqued for fails to address gaps, such as the lived experiences of trans, gender fluid and nonbinary people. Tate (2012) suggests that being assigned at birth to a specific gender category should not be privileged over self-categorization later in life. Thus, an exploration of the ways young trans, gender fluid or nonbinary people “do” gender and “be” gender is crucial in understanding how all young people engage with porn.

### Conclusion

In the past few years more research has made visible young people’s experiences with porn and the meanings they attach to these experiences. This article offers insights into the ways young people understand and perform gender in regards to watching/ not watching, talking/ not talking about porn. The young participants both performed and resisted dominant gendered narratives on porn through their actions—for example, watching, or not watching porn, and interactions—discussing or not discussing porn with their peers. Sex education could provide fertile ground to critical explore the structural contexts and dominant discourses which impacted the meanings young people attached to their experiences with porn and interactions with peers about porn.
